# Rectal cancer lexicon 2023 revised and updated consensus statement from the Society of Abdominal Radiology Colorectal and Anal Cancer Disease-Focused Panel

**DOI:** 10.1007/s00261-023-03893-2

**Published:** 2023-05-05

**Authors:** Sonia Lee, Zahra Kassam, Akshay D. Baheti, Thomas A. Hope, Kevin J. Chang, Elena K. Korngold, Melissa W. Taggart, Natally Horvat

**Affiliations:** 1 Radiological Sciences, University of California, Irvine, Irvine, CA USA; 2 Department of Medical Imaging, Schulich School of Medicine, St Joseph’s Hospital, Western University, London, ON N6A4V2 Canada; 3 Department of Radiology, Tata Memorial Hospital and Homi Bhabha National Institute, Mumbai, Maharashtra India; 4 Department of Radiology and Biomedical Imaging, University of California, San Francisco, San Francisco, CA USA; 5 Department of Radiology, Boston University Medical Center, Boston, MA USA; 6 Department of Radiology, Oregon Health & Science University, Portland, OR USA; 7 Department of Pathology, The University of Texas MD Anderson Cancer Center, Houston, TX USA; 8 Department of Radiology, Memorial Sloan Kettering Cancer Center, New York, NY USA; 9 University of California at Irvine, 101 The City Dr. S, Orange, CA 92868 USA

**Keywords:** Rectal cancer, Rectal adenocarcinoma, Gastrointestinal tract, MRI, Oncology

## Abstract

The Society of Abdominal Radiology’s Colorectal and Anal Cancer Disease-Focused Panel (DFP) first published a rectal cancer lexicon paper in 2019. Since that time, the DFP has published revised initial staging and restaging reporting templates, and a new SAR user guide to accompany the rectal MRI synoptic report (primary staging). This lexicon update summarizes interval developments, while conforming to the original lexicon 2019 format. Emphasis is placed on primary staging, treatment response, anatomic terminology, nodal staging, and the utility of specific sequences in the MRI protocol. A discussion of primary tumor staging reviews updates on tumor morphology and its clinical significance, T1 and T3 subclassifications and their clinical implications, T4a and T4b imaging findings/definitions, terminology updates on the use of MRF over CRM, and the conundrum of the external sphincter. A parallel section on treatment response reviews the clinical significance of near-complete response and introduces the lexicon of “regrowth” versus “recurrence”. A review of relevant anatomy incorporates updated definitions and expert consensus of anatomic landmarks, including the NCCN’s new definition of rectal upper margin and sigmoid take-off. A detailed review of nodal staging is also included, with attention to tumor location relative to the dentate line and locoregional lymph node designation, a new suggested size threshold for lateral lymph nodes and their indications for use, and imaging criteria used to differentiate tumor deposits from lymph nodes. Finally, new treatment terminologies such as organ preservation, TNT, TAMIS and watch-and-wait management are introduced. This 2023 version aims to serve as a concise set of up-to-date recommendations for radiologists, and discusses terminology, classification systems, MRI and clinical staging, and the evolving concepts in diagnosis and treatment of rectal cancer.

**Graphical abstract:**

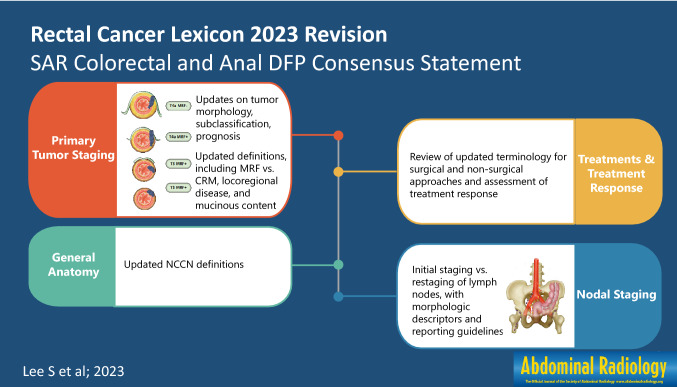

## Introduction

Standardized lexicons in radiology facilitate and improve the agreement between radiologists and referring clinicians as well as enhance multicenter research. Revisions to these standardized terminologies are dependent on the evidence-based imaging research to date, expert consensus, national and international clinical practice guidelines, and evolution in the understanding of underlying disease processes. This paper provides an overview of the updates to the 2019 Society of Abdominal Radiology (SAR) Colorectal and Anal Cancer Disease-Focused-Panel (DFP) lexicon which is used frequently in rectal cancer [1, 2], and reflects the consensus of the SAR Colorectal and Anal Cancer DFP. Staging recommendation is based on American Joint Committee on Cancer (AJCC) Cancer Staging System Protocol 8th version as 9th version has not been published at the time of submission. Previously used lexicon categories have been maintained, as follows: (1) Primary tumor staging, (2) Nodal staging, (3) Treatment response, (4) Anal canal anatomy, (5) General anatomy, and (6) Treatment.

## Primary tumor staging

### Summary of update 1

The previous statement that “MRI frequently cannot distinguish between T1 and T2 tumors” has been updated to include the use of the submucosal enhancing stripe sign for this purpose. T1 subclassification based on the Kudo and Kikuchi classification used in histology has been introduced as the latter is included in the National Comprehensive Cancer Network (NCCN) guidelines; however, its use in radiology reporting is not recommended because magnetic resonance imaging (MRI) is limited for such classification. The prognostic implication of T3 subcategories, MRI findings supporting T4a classification, specific guidance regarding T4b classification, and the recommended approach for anal sphincter involvement description have been added.

**Fig. 1:**
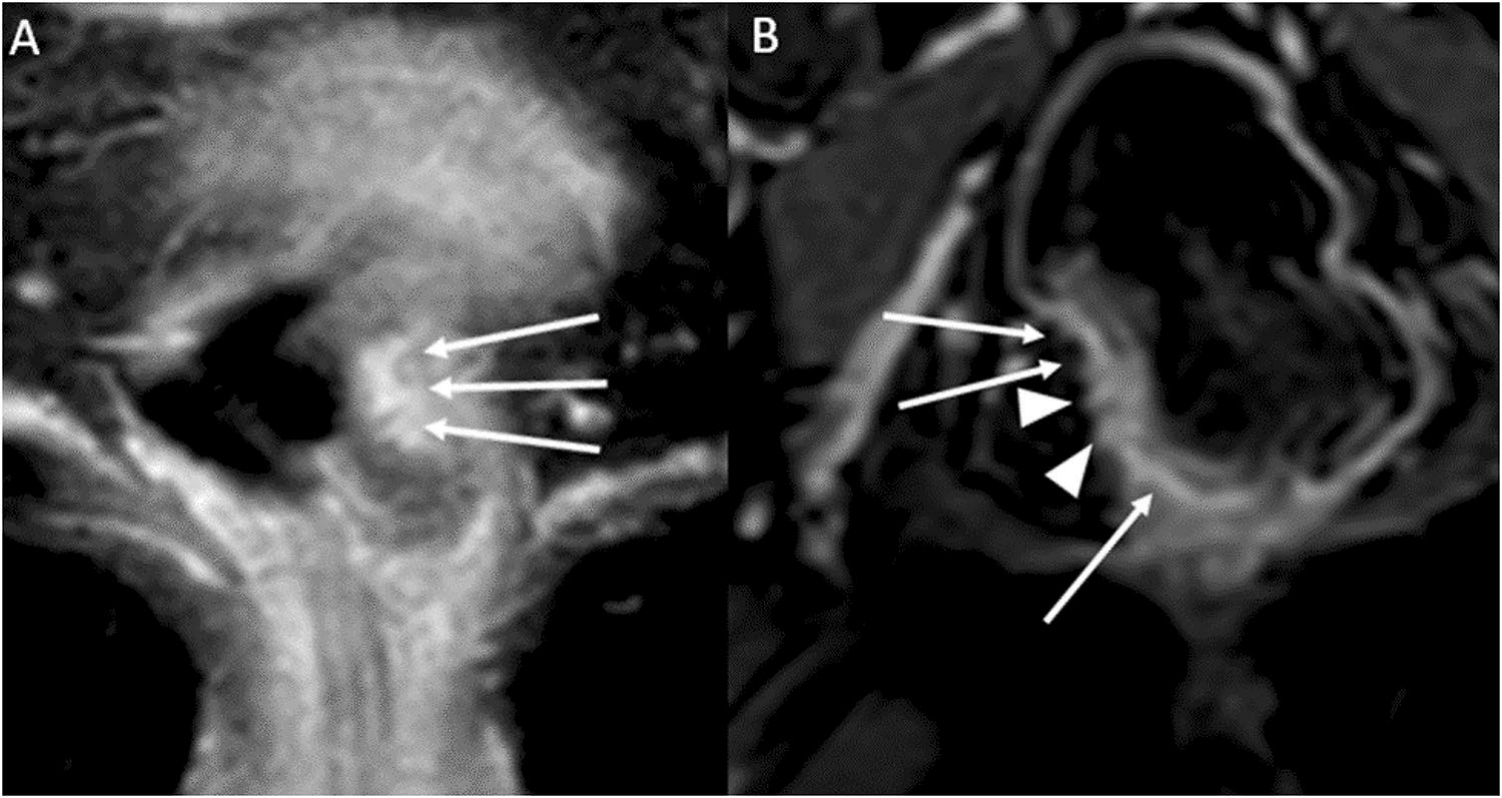
Examples of submucosal enhancing stripe (SES). **A** T1 tumor demonstrates intact SES throughout its base (long thin arrows). **B** T2 tumor with disrupted SES. The submucosal stripe is intensely enhancing at the periphery of the tumor(long thin arrows). However, it is interrupted at the central base (arrowheads), suggestive of invasion through the submucosal layer [6]

**Fig. 2:**
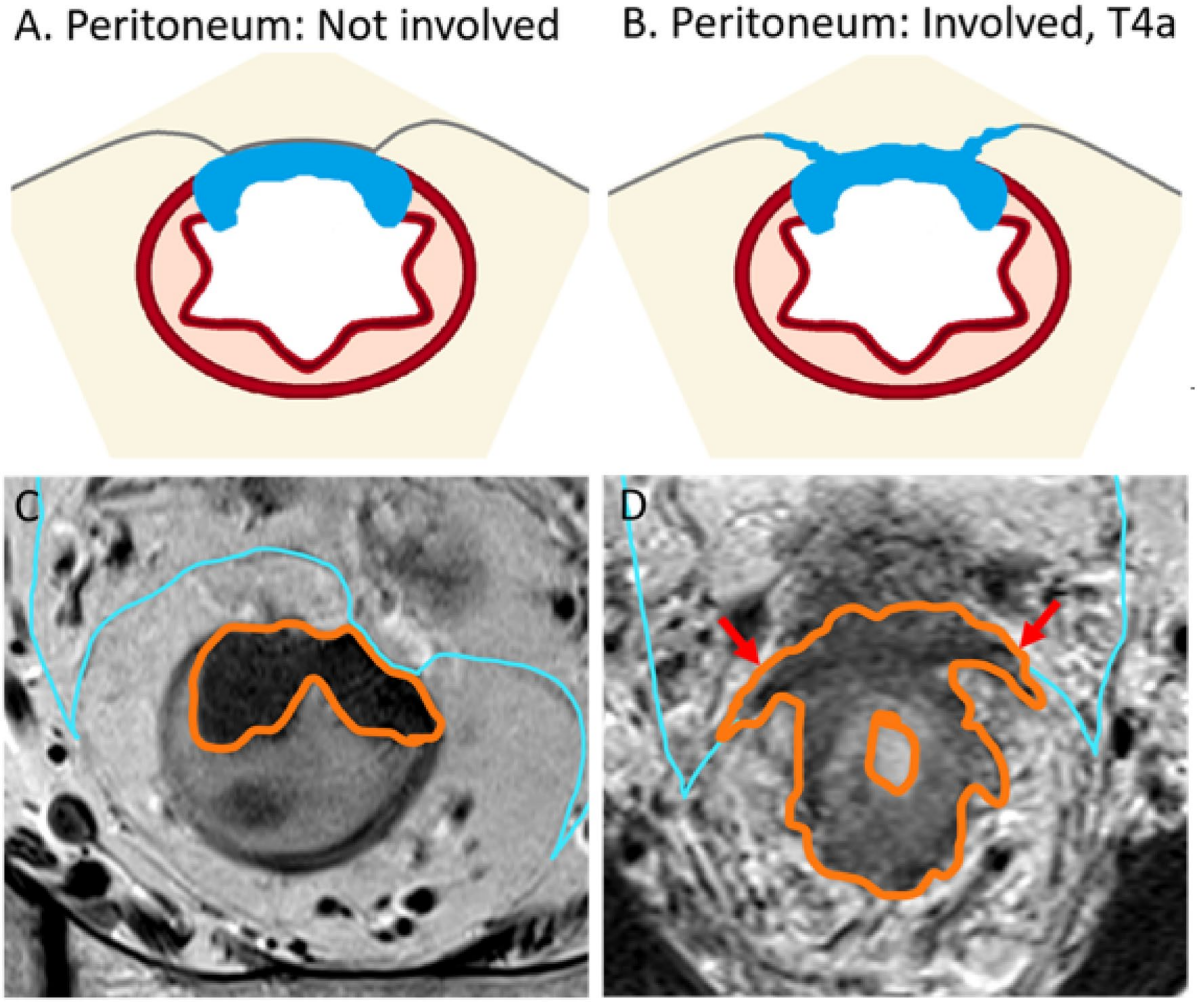
Rectal cancer without and with peritoneal involvement at the anterior peritoneal reflection illustrations and MRI examples. Accurate categorization will prevent unnecessary overtreatment which has been reported to occur in greater than 10% of MRI interpretation [68]. Rectal tumor abutting the peritoneum alone should not be considered peritoneal invasion. Illustration **A** demonstrates tumor (blue) abutting the peritoneum without involvement. Correlating MRI T2-weighted oblique axial image (**C**) demonstrates high rectal tumor (orange outline) partially enveloped by peritoneum (light blue thin line) anteriorly without peritoneum thickening, with no evidence of invasion. Illustration B demonstrates nodular peritoneal thickening at the tumor base(blue) consistent with T4a. **B** MRI T2W axial image (**D**) demonstrates annular tumor (orange outline) peritoneum nodular thickening extending laterally along the anterior peritoneal reflection (red arrows).T3 tumor of the upper rectum may be considered appropriate for primary surgical resection, if otherwise low risk, with no lymph node involvement, EMVI, or mesorectal involvement [15]. T4a tumors are associated with peritoneal metastasis

- *T-Classification* (For MRI) Tx: primary tumor cannot be assessed; T0: no visible primary tumor; T1: tumor extends to involve the submucosa; T2: tumor extends to involve the muscularis propria; T3: tumor extends beyond the muscularis propria to involve mesorectal fat (T3a-d based on depth of extramural invasion in mm); and T4: tumor infiltrates/invades the peritoneum (T4a) or other pelvic organs and structures (T4b).
  - T1 may be subclassified into sm1, sm2, and sm3, reflecting the depth of submucosal (sm) invasion in one-third increments, per the Kudo and Kikuchi classification [3–5]. Notably, the Kudo and Kikuchi classification is used to classify hollow viscus neoplasms in histologic reporting, and T1 sm1 is associated with very low risk of lymph node metastasis.
  - T1 and T2 are combined in the SAR DFP reporting template as a single category of T1/2, given that the differentiation between the two is difficult using MRI. For the differentiation of T1 and T2, endoscopic ultrasound performs better than MRI, especially if the lesion is very small and flat. If T1 and T2 differentiation is attempted, careful evaluation of the submucosal architecture or muscularis propria involvement on T2 high-resolution sequences, or submucosal enhancing stripe evaluation with postcontrast sequences may be helpful [6] (Fig. 1).
  - T3 may be grouped into two prognostic categories, as follows: *T3 a/b (good prognosis)* with up to 5 mm extramural invasion; and *T3 c/d (higher risk of local recurrence)* with more than 5 mm invasion beyond the muscularis propria. Any T3 substage with mesorectal fascia (MRF) involvement is also associated with a higher risk of local recurrence [7–9].
  - T4a or peritoneal invasion is suspected on MRI when there is altered signal intensity, thickening, or nodularity of the peritoneum, but not when there is abutment of the peritoneum by tumor (Fig. 2).
  - T4b includes the involvement of adjacent pelvis organs, including the uterus, ovaries, vagina, prostate, seminal vesicles, bladder, ureters, ureter, bone, and skeletal/striated muscular structures, such as the obturator, piriformis, ischiococcygeus, levator ani, and puborectalis muscles. Additionally, recent expert consensus suggested assigning a T4b category if the tumor involves the following structures: extramesorectal vessels, sciatic or sacral nerves, sacrospinous/sacrotuberous ligaments, and soft tissue beyond the mesorectum, such as fat of the obturator, iliac, or ischiorectal space [10]. Of note, involvement of the MRF, anterior peritoneal reflection, internal anal sphincter, or intersphincteric space does not constitute T4b [10].
  - Regarding anal sphincter, the 8th edition of the American Joint Committee on Cancer (AJCC) staging manual does not specify whether external sphincter involvement should be considered T4b [11]. However, a recent multidisciplinary international expert consensus and the College of American Pathologists expert consensus both define external anal sphincter involvement as T4b [10, 12]. The SAR DFP has not reached a consensus as to whether external anal sphincter involvement should be characterized as T4b. There is clear consensus, however, that in reporting the anal involvement, a specific description of the level of involvement, such as the level of involvement of the internal sphincter, intersphincteric plane, or external sphincter, and the location/length (upper/mid/distal) of the involvement should be communicated to the surgeon, to help select the appropriate surgical option [13]. In addition, if a tumor is classified as T4b, each structure involved should be clearly specified, and the reporting pattern should be consistent within the institution to avoid confusion [1].

### Summary of update 2

Locally advanced rectal cancer terminology was previously included in the “Treatment” category but is now included in the “Primary tumor staging” category of the lexicon. Additionally, both the United States NCCN and European Society for Medical Oncology (ESMO) definitions of locally advanced rectal cancer are now included.

- *Locally advanced rectal cancer* Locally advanced rectal cancers commonly refer to tumors that would likely benefit from neoadjuvant therapy, which is performed to reduce the risk of a positive resection margin or when total mesorectal excision (TME) may not achieve curative resection. In the United States, based on NCCN guidelines, such tumors may include T3 or T4, or any T N + [14]. Of note, low-risk T3 N0 upper rectal tumor may be considered appropriate for primary resection, and not locally advanced[15]. In Europe, based on ESMO guidelines, tumors that are (1) T3c/d, (2) very low rectum, (3) with extramural vascular invasion, (4) any T3 with the MRF involved, (5) T4b, (6) levator threatened, or (7) with lateral lymph node involved are considered locally advanced rectal cancers [16, 17]. The European approach considers T3a/b with no other high-risk feature appropriate for upfront total mesorectal resection, as it has demonstrated similarly low positive resection margin rate and favorable prognosis as T2 [7, 18].

### Summary of update 3

As the SAR DFP baseline template has been updated to use the term MRF rather than circumferential resection margin (CRM), that change to the lexicon has been made accordingly [13, 19]. Detailed description of the criteria used to evaluate MRF involvement are specified.

**Fig. 3:**
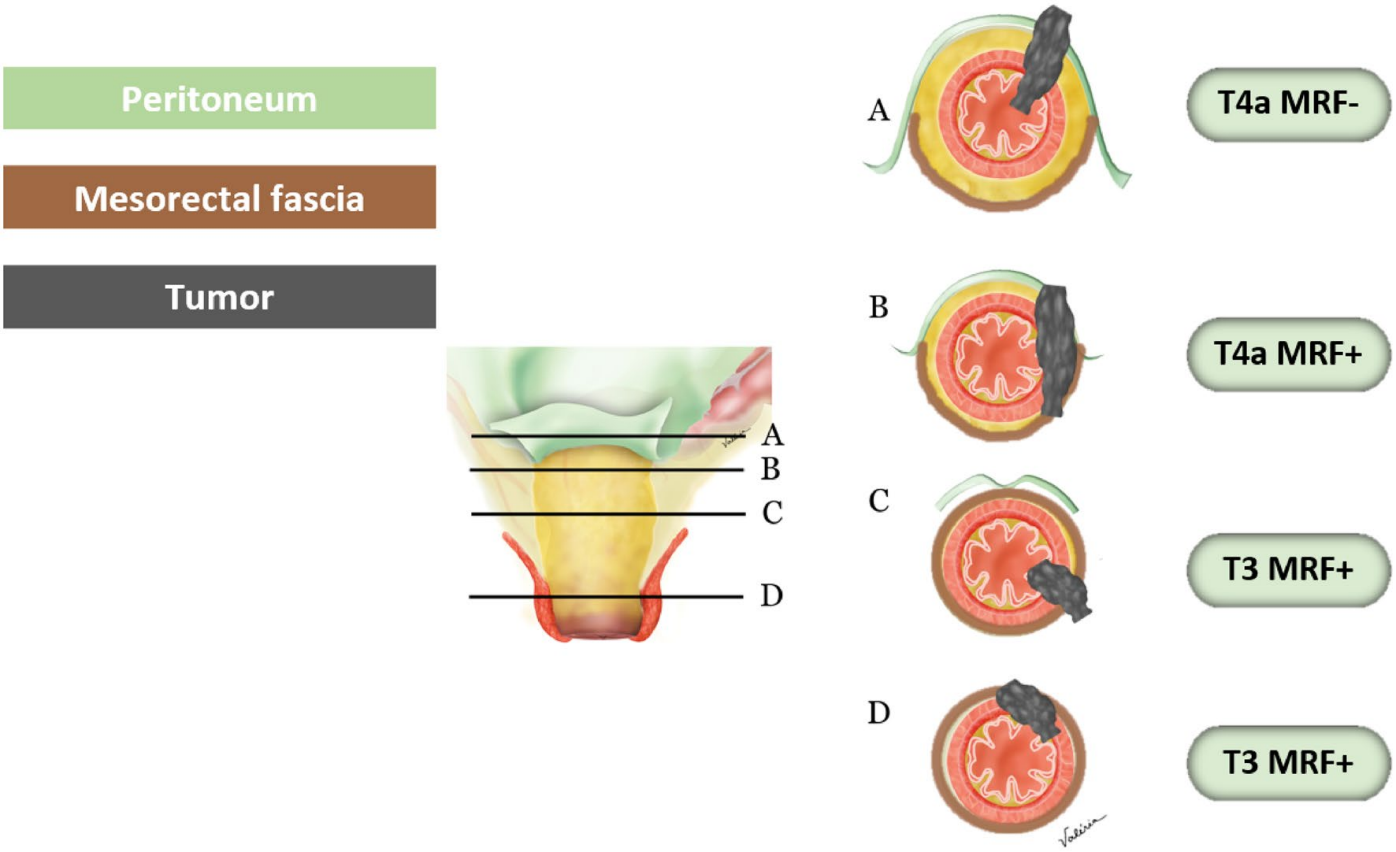
Peritoneal and mesorectal fascia (MRF) involvement. **A** Tumor of the anterior wall above the anterior peritoneal reflection involving the peritoneum. **B** Tumor above the anterior peritoneal reflection on the lateral wall extends anteriorly to involve the peritoneum and posteriorly to involve the MRF. **C** Tumor at the level of the anterior peritoneal reflection extends posterior and laterally, involving the MRF only. **D** Tumor below the anterior peritoneal reflection, involving the MRF anteriorly

- *MRF* The MRF is the anatomic fascial plane used to guide TME. The CRM may differ from MRF depending upon the surgical approach, for example, a surgeon may attempt to excise more extramesorectal fat beyond the MRF in a location suspected to be involved/threatened or perform an abdominoperineal resection or pelvic exenteration. In the preoperative staging report template, the DFP has therefore elected to use the term MRF which is an anatomic term rather than CRM which is the operative surgical margin dependent on the surgical approach.
- *MRF status* The MRF status depends on the shortest distance between the MRF and the outermost part of the rectal tumor, including extramural vascular invasion, tumor deposits, or capsule disrupted positive lymph nodes [20]. Lymph nodes with an intact capsule are not considered involved as they are not associated with increased local recurrence rates [21, 22]. The SAR DFP template uses a three-tiered system for MRF status: “involved” for a distance < 1 mm, “threatened” for 1–2 mm, and “clear” for > 2 mm [13]. However, a recent international multidisciplinary expert consensus recommended simplification into only two tiers: “involved” for < 1 mm, and clear for ≥ 1 mm [10]. SAR DFP has not reach agreement on whether to endorse this recent consensus recommendation. On resected specimens at pathology, for tumor distances to CRM of < 1 mm, 1–2 mm, and > 2 mm, the local recurrence rates were 36%, 16%, and 6%, respectively [23]. However, this measured distance was between the tumor and the resection margin, not the MRF. Moreover, trials have shown that a 1-mm threshold achieved excellent results, e.g., local recurrence rates of 3.3% at 5 years in MERCURY (Magnetic Resonance Imaging in Rectal Cancer European Equivalence Study) and 2.2% at 3 years in OCUM (Optimierte Chirurgie Und MRT—optimized surgery and MRI-based multimodal therapy), and a 4.2% positive CRM rate in QuickSilver [8, 18, 24]. As the MRF runs over the surface of the levator ani inferiorly, low rectal tumors contacting or located within 1 mm of the MRF over the surface of the levator ani are considered MRF-involved tumors (Fig. 3).

### Summary of update 4

Terms describing tumor morphology have been separated from terms describing tumor composition (such as the mucinous component) in the updated lexicon. Additional guidance on tumor morphology, including the clinical significance of various morphologies, has been included to reflect the most current published data.

- *Annular/circumferential* Annular/circumferential tumors involve the entire circumference of the rectal lumen.
- *Partly annular/semicircumferential* Partly annular/semicircumferential tumors only partially involve the circumference of the lumen, often greater than 90° but less than 360°. These tumors often demonstrate rolled-up edges at the margin and central ulceration. Frequently, the deepest invasion occurs at the point of maximal mural ulceration [25].
- *Polypoid* Polypoid lesions are intraluminal tumor protrusions attached to the rectal wall with a broad base (sessile) or stalk (pedunculated) and typically involve less than 90° of the luminal circumferences. Vessels may be visible as flow voids within the stalk or pedicle on T2-weighted imaging (T2WI). Compared to annular or semiannular morphologies, a polypoid morphology is often associated with lower clinical stages of a neoplasm, such as adenoma, intramucosal adenocarcinoma (involvement of lamina propria, previously referred to as carcinoma in-situ), or T1/2 if malignant [25].

### Summary of update 5

The characterization of tumor composition is important, as mucin-containing rectal carcinomas are associated with microsatellite instability, BRAF and KRAS mutations, and MUC-2 overexpression—genetic conditions that may have treatment implications. Although the World Health Organization’s definition of these tumors is based on the percentage of mucin identified on histology, it is difficult to quantify histologic percentage on imaging. Therefore, our recommendation is to document the degree of abundance into categories of (1) no mucin, (2) some mucin, or (3) mostly mucin [13].

- *Mucin-containing rectal cancer* Mucin-containing rectal cancers demonstrate high signal intensity on T2WI and may present in two possible forms [26]. The more common subtype is typical mucinous carcinoma, which is composed of greater than 50% extracellular mucin on histology. The less common subtype is signet ring carcinoma, which contains cells with intracytoplasmic mucin. Both subtypes, when combined, account for approximately 5–10% of all rectal cancers in the United States. Both subtypes are also associated with presentation at a younger age and aggressive histology [27, 28].
- *Non-mucinous rectal cancer* Non-mucinous rectal cancers are rectal adenocarcinomas that demonstrate intermediate signal intensity on T2WI; they constitute the majority of rectal adenocarcinomas in the United States [29].

## Nodal staging

### Summary of the updates

**Fig. 4:**
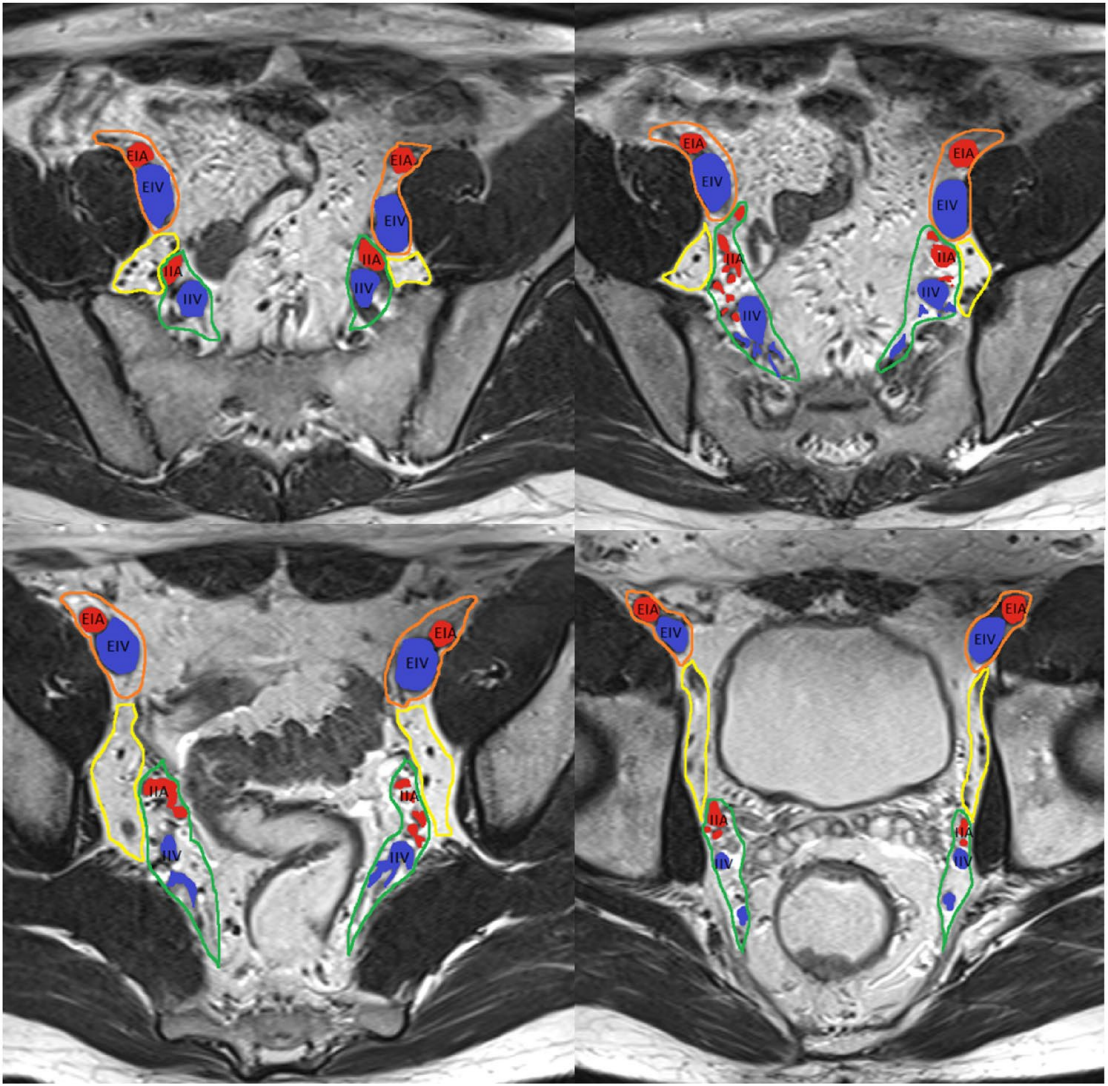
Pelvis side wall node anatomy, from upper to lower pelvis. External iliac arteries (EIA, red), external iliac vein (EIV, blue), internal iliac arteries (IIA, red), and internal iliac veins (IIV, blue), and their relationship with the external iliac lymph node region (orange), internal iliac lymph node region (green), and obturator lymph node region (yellow) are depicted. At the level of the obturator muscle, nodes medial to the internal iliac artery are internal iliac lymph nodes (region outlined in green). Lymph nodes lateral to the internal iliac artery within the yellow boundary are obturator lymph nodes [33]

**Fig. 5:**
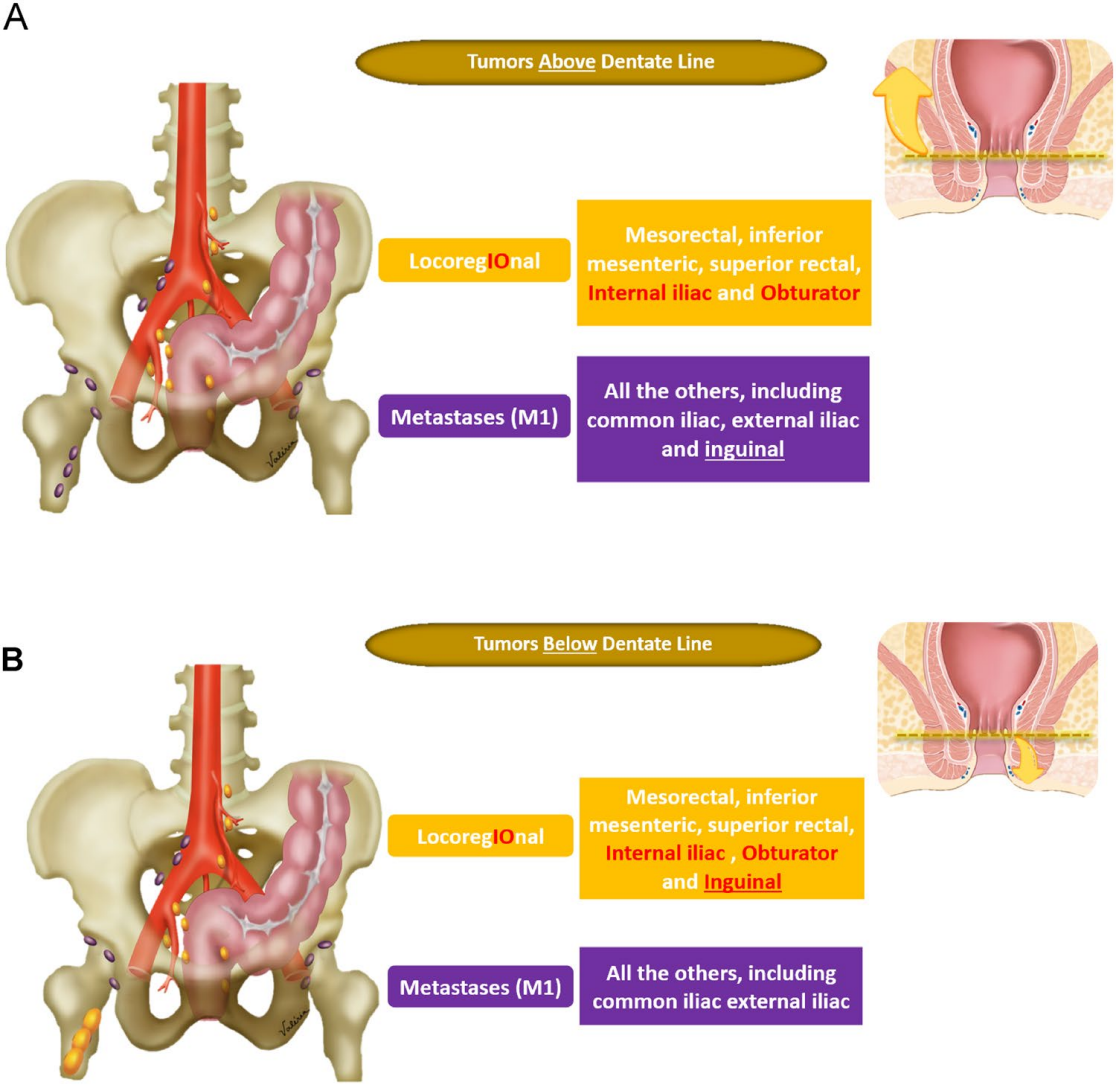
Pelvic nodal drainage pathway in relation to the dentate line. **A** For a tumor above the dentate line, the locoregional nodes (colored in gold) include the inferior mesenteric, superior rectal, internal iliac, obturator, and mesorectal nodes. Non-locoregional nodes (colored in purple) include the common iliac, external iliac, and inguinal nodes. **B** For a tumor below the dentate line, the locoregional nodes (colored in gold) also include the inguinal lymph nodes. Non-locoregional nodes (colored in purple) include the common iliac and external iliac nodes

**Fig. 6:**
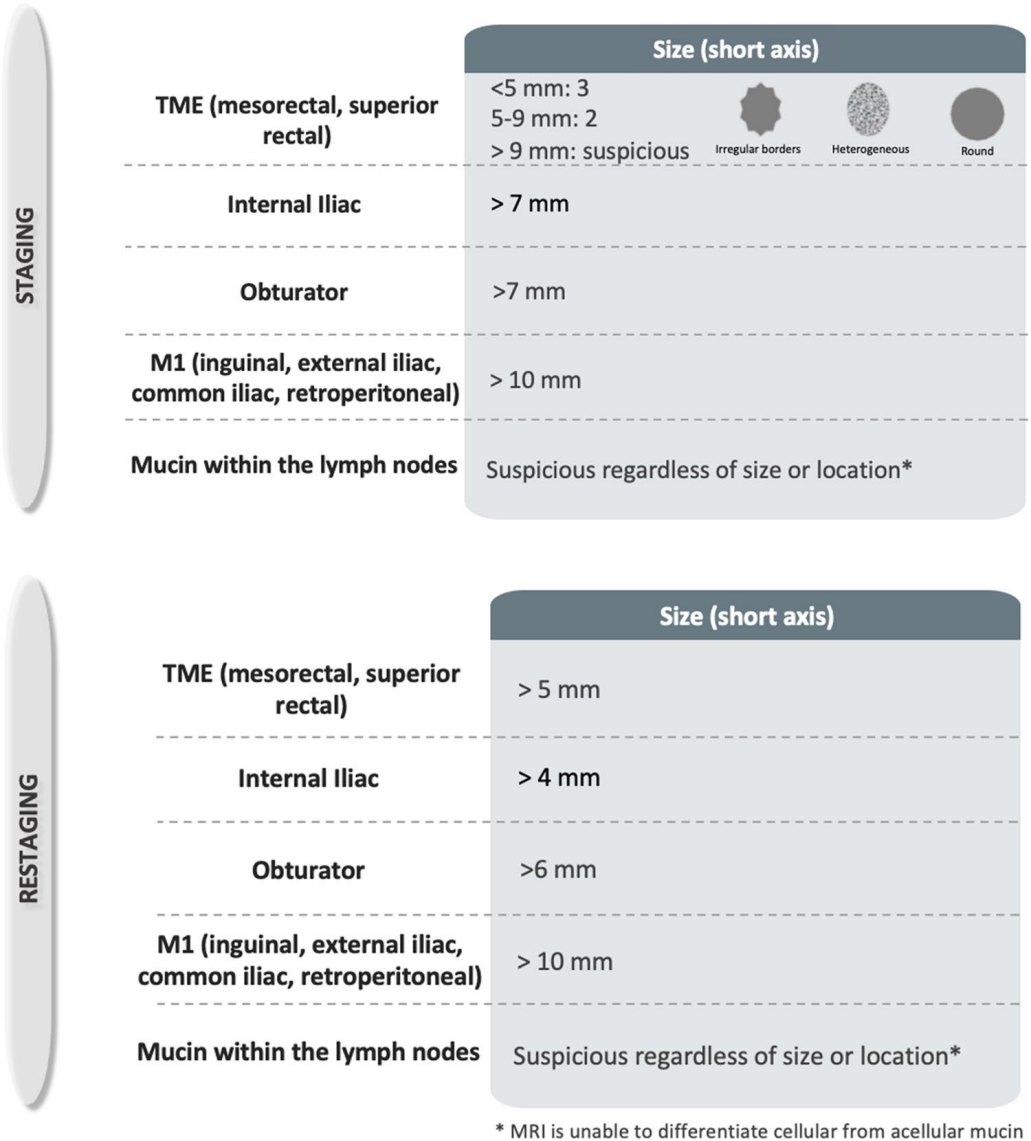
Criteria for suspicious lymph nodes on initial staging and restaging after neoadjuvant therapy

Recent studies suggest that tumor deposits, which are considered N1c according to TNM classification, are more strongly associated with poor prognosis compared with lymph node involvement [30]. Therefore, morphologic descriptors to differentiate tumor deposits from lymph nodes have been included [31]. Clarification on rectal cancer location in relation to the dentate line, and changes in the categorization of inguinal lymph nodes between locoregional or non-locoregional nodes have been specified [32]. Regarding lateral lymph nodes, which Dutch criteria are not applicable, size criteria associated with a higher likelihood of local recurrence have been included [33]. Lymph node restaging criteria for suspicious nodes have also been included [34, 35] and the importance of high-quality imaging in accurate lymph node assessment has been added [36, 37]. Figures 4, 5, and 6 have been added to improve understanding.

- *Locoregional lymph nodes* Mesorectal, superior rectal, and inferior mesenteric nodes (superior to the take-off of the left colic artery from the inferior mesenteric artery), as well as internal iliac and obturator lymph nodes are considered locoregional lymph nodes in the setting of rectal cancer [38] (Fig. 4). If the tumor extends below the dentate line, inguinal lymph nodes are also considered locoregional lymph nodes [32] (Figs. 5).
  - *Initial staging criteria for suspicious locoregional mesorectal, superior rectal, and inferior mesenteric lymph nodes* The Dutch Criteria have been adopted by both the European Society of Gastrointestinal and Abdominal Radiology (ESGAR) and the SAR Colorectal and Anal Cancer DFP; these criteria include the short-axis dimension and morphologic characteristics including irregular borders, heterogeneous signal intensity, and round shape [34, 39]. If a locoregional lymph node is *greater than* 9 mm *in short axis,* it is considered suspicious, regardless of morphology; if 5–9 mm* in short axis*, then two morphologic criteria are required; and if < 5 mm* in short axis*, then 3 criteria are required.
  - *Initial staging criteria for suspicious locoregional lateral pelvic side lymph nodes* For internal iliac and obturator lymph nodes, a size of > 7 mm in the short axis is required for these nodes to be considered suspicious. Notably, size criteria from the multicenter lateral node study [40] are applicable in the setting of T3/4 tumors located < 8 cm from the anal verge but their application should be limited in the setting of early T1/2 tumors or high rectal tumors, as the likelihood of lateral nodal spread in T1/2 tumors is very low.
  - *Restaging criteria after neoadjuvant therapy for suspicious locoregional mesorectal, superior rectal, and inferior mesenteric lymph nodes* After neoadjuvant chemoradiotherapy (CRT), the Dutch criteria are no longer applicable. Instead, size criteria are used to identify lymph nodes suspicious for persistent involvement. If a mesorectal, superior rectal, or inferior mesenteric node measures > 5 mm, it should be considered suspicious (Fig. 6). Although the complete loss of diffusion restriction in lymph nodes after neoadjuvant treatment is uncommon, it suggests treatment response [35].
  - *Restaging criteria for suspicious locoregional lateral pelvic side lymph nodes* If an internal iliac lymph node remains > 4 mm or if an obturator lymph node is > 6 mm after neoadjuvant CRT, these are considered suspicious as they are associated with local recurrence, again in the setting of tumors < 8 cm from the anal verge, according to multicenter international study by lateral node study consortium (Fig. 6) [33].
- *Non-locoregional/distant lymph nodes* External iliac, common iliac, paraaortic, and inguinal nodes (if the rectal cancer is above the dentate line) are considered non-locoregional/distant lymph nodes (metastatic disease, M1) in the setting of rectal cancer. Non-locoregional lymph nodes may be considered suspicious if they measure > 10 mm in the short axis. However, the location of the tumor, its expected drainage pattern, and malignant features such as parenchymal signal abnormality, abnormal lymph node border, asymmetry, and spherical shape should also be considered as lymph node short-axis enlargement is not a specific indicator of malignancy [41]. This is especially important in the evaluation of elongated posterior/caudal/medial external iliac region lymph nodes as their involvement is extremely rare, hence their being excluded from consideration in a multicenter lateral node study [34].
- *N classification* If N classification is made on pelvic MRI, the DFP recommends using “N +” for abnormal locoregional lymph nodes and/or tumor deposits and “N −” for the absence of locoregional nodal disease, rather than specifying the N classification into N0, N1a, N1b, N1c, or N2.[13]. Although, the specificity of N classification has significantly improved with the Dutch criteria, its sensitivity remains mediocre especially in small lymph node involvement, and therefore accurate number assessment is difficult. In addition, evidence of imaging performance in differentiation between tumor deposit and malignant lymph node is limited.
- *Heterogeneous* Classically, this term is used to describe lymph nodes with internal elements of variable signal intensity. Although the term heterogeneous is used, it would be more accurate to describe this as abnormal parenchymal signal, as mucinous involvement should also be considered abnormal. Mucinous composition may be interposed between solid tissue with heterogenous overall signal, or if it is abundant, the lymph node may appear homogenously T2 hyperintense. If this characteristic is applied, according to the MERCURY study, it has a sensitivity of 48% and a specificity of 99% for tumor involvement [42] (Fig. 5).
- *Irregular border* An irregular border is seen when there is transgression of the lymph node capsule or when there are tumor deposits, and may be seen as an angulated or spiculated margin [20]. Both high spatial resolution and low image noise on T2WI are important to allow adequate assessment of this feature and the heterogenous signal mentioned above. If this characteristic is applied, according to the MERCURY study, it has a sensitivity of 75% and a specificity of 98% for tumor involvement [42] (Fig. 6).
- *Tumor deposit* Tumor deposit is defined as a tumor nodule with no associated lymph node tissue, in the drainage area of the primary tumor on histology [43]. Tumor deposits may originate from discontinuous tumor spread, lymphatic spread, venous invasion, or a totally replaced lymph node. It is a greater indicator of poor prognosis than lymph node involvement [10, 44]. Recent studies have suggested that contiguity with vein on two orthogonal planes, tumor tissue tapering into the vein (comet tail appearance), and irregularity in shape are MRI features more indicative of tumor deposits rather than lymph nodes [30, 31].

## Treatment response

### Summary of the update

New terms including near-complete response (nCR), incomplete response (iCR), tumor regrowth, and recurrence have been added. The SAR Colorectal and Anal Cancer DFP recommends treatment response at restaging to be categorized into (1) complete (CR)/nCR, (2) iCR, or (3) no response. CR and nCR may be observed on MRI [45]. Patients with iCR or no response on MRI are not eligible for watch-and-wait management as both are considered a poor response. As treatment response is assessed not only by MRI, but also clinical examination and endoscopy and there may be discrepancy between the assessments, prefix may be added for clarification such as mrCR.

- *CR/nCR* CR and nCR entail complete or near-complete resolution of T2 intermediate signal, respectively, with significant regression but incomplete resolution of diffusion restriction after neoadjuvant treatment [46]. Most cases of CR and nCR, ranging from 73 to 99%, will be converted to CR at short-term follow-up 6–12 weeks after neoadjuvant treatment [47]. If tumor signal or diffusion restriction persists after one or two short-term follow-up assessments, the case should be converted to iCR and considered unfit for observation. As it appears safe to defer surgery for both CR and nCR cases if closely monitored in hands of those with expertise, they are grouped into a single category [17, 48].
- *iCR* iCR is defined as a decrease in the volume of the primary tumor but with definite residual tumor, evident by residual diffusion restriction in the tumor above background level and residual T2 intermediate signal tumor. Although the term iCR is sometimes used interchangeably with “partial response,” the SAR Colorectal and Anal Cancer DFP recommends the term “incomplete” [17, 48].
- *Regrowth* Regrowth entails the re-emergence of tumor after CR, specifically in patients who underwent watch-and-wait management. Regrowth is characterized by the re-emergence of a characteristic intermediate signal tumor on T2W images, diffusion restriction, scar thickening, or heterogenous signal intensity emerging from a previously homogenous low signal intensity scar [49]. Local regrowth refers to the detection of a tumor involving the bowel wall only, while locoregional regrowth refers to the detection of tumor involving either the bowel wall, mesorectum, and/or pelvic organs [50]. Tumor re-emergence may also be described as luminal regrowth if primary tumor, and nodal regrowth, if lymph node involvement, respectively.
- *Recurrence* Recurrence entails the detection of tumor after local excision or TME. Local recurrence refers to involvement of bowel wall only, while locoregional recurrence refers to involvement of either bowel wall, mesorectum, and/or pelvis organs [50].

## General anatomy

### Summary of the update

In consideration of varied body types and sizes, the NCCN has defined the upper border of rectum as a virtual line from the sacral promontory to the upper border of pubic symphysis. Additionally, the sigmoid colon take-off has been suggested by an expert consensus group as the best landmark for determining the rectosigmoid junction [51]. These new definitions have been added to the lexicon. A description of submucosa evaluation using the submucosal enhancing stripe sign, and the new term “retrorectal space” have also been added.

- *Rectum* The upper margin is defined by the NCCN as below a virtual line from the sacral promontory to the upper edge of the symphysis as determined by MRI, which roughly correlates to 15 cm of large bowel immediately superior to the anal verge. The lower rectal margin is at the anorectal junction. Anatomically, the rectum extends only to the dentate line which is inconsistently visualized at MRI.
- *Rectosigmoid junction* The rectosigmoid junction refers to the transition between the rectum and the sigmoid. There have been multiple anatomic landmarks and different measurements from the anal verge suggested as appropriate transition points. Recently, a multidisciplinary expert group has issued a consensus that the sigmoid take-off is the most appropriate anatomic landmark [52, 53].
- *Sigmoid take-off* An anatomic landmark of the rectosigmoid junction, it is seen as the part of the colon that turns away from the sacrum and extends anteriorly, usually a few centimeters above the anterior peritoneal reflection [52].
- *Retrorectal space* Broadly, the retrorectal space refers to the space between the posterior rectal wall and ventral surface of the sacrum/coccyx/posterior pelvic floor [54]. Others use this term more strictly, referring only to the lower aspect of that space [55]. The most specific use of this term would be to refer the space defined by the posterior MRF anteriorly, the lower sacrum and coccyx posteriorly, the levator ani inferiorly, the coccygeal muscle laterally, and the peritoneal reflection superiorly [56]**.**
- *Submucosa* The submucosa is the middle rectal layer which is hyperintense on T2WI. The layer’s rich vascularity may be used to differentiate early T1 and T2 tumors via the submucosal enhancing stripe sign if intravenous contrast is used [6] (Fig. 1). The submucosal enhancing stripe sign is based on data from a single institution, and therefore should be used with caution. SAR DFP stance that EUS performs superior to MRI in small/flat T1/2 tumor differentiation in general and default intravenous contrast use is not recommended are unchanged.
- *Upper rectum* By measurement, the upper rectum is 10–15 cm proximal to the anal verge; by anatomic landmarks, it is roughly from the anterior peritoneal reflection to the sigmoid take-off. It is partially enveloped by the peritoneum anteriorly.

## Miscellaneous terms

### Summary of the update

High-resolution T2WI is critical for the evaluation of the rectum; a more specific description of what constitutes high resolution has been added. Diffusion-weighted imaging (DWI) has also been included to the lexicon.

- *DWI* DWI is an increasingly used MRI sequence in rectal cancer evaluation. Use of a high *b*-value (*b*-value of at least 800) and the inclusion of apparent diffusion coefficient (ADC) mapping are recommended. Visual identification of persistent high signal on high B value diffusion restriction sequence, and correlating low signal on ADC map is helpful in differentiation of tumor signal from T2 shine through (high signal on both diffusion restriction sequences and ADC map, see with mucin or high water content) and T2 dark through (high signal on both diffusion restriction sequences and ADC map, seen with fibrous scar). DWI is helpful for the assessment of treatment response at restaging, for tumor localization, and for lymph node localization; potentially, DWI may also be used as a fat-saturated sequence in mucin-containing tumors, to help detect high signal tumor that may be similar in signal to background mesorectal or extraperitoneal fat. However, its value remains limited for T or N classification and for the assessment of treatment response in mucinous rectal tumors [57–59].
- *High-resolution T2WI* High-resolution T2WI is the cornerstone of rectal MRI. High resolution is achieved by a field of view (FOV) and matrix allowing a plane resolution of approximately 0.6 × 0.6 mm; for example, for a 16-cm FOV, the matrix is 256 × 256. If the slice thickness is set at 3 mm, this achieves a voxel size of 1.08 mm^3^ [36, 37, 60]. Of the various T2 sequences, the oblique axial sequence perpendicular to the tumor invasion plane is the most important sequence and must be obtained in high resolution.

## Treatment

### Summary of the update

New terminology have been added including organ preservation, total neoadjuvant treatment (TNT), watch-and-wait management, radiation therapy options of long-course versus short-course chemoradiotherapy, and transanal minimally invasive surgery (TAMIS). Specific indications for various transanal excision methods have also been added to help tailor the radiology report to referring surgeons’ needs.

- *Organ preservation* This is a term for retaining the rectum, owing to no radical TME; no locoregional regrowth unless amenable to limited, curative salvage surgery by local excision; and no permanent stoma (including a never reversed protective stoma, or a stoma owing to toxicities and/or poor functional outcomes) [50].
- *Radiation* Radiation therapy can be part of neoadjuvant treatment as conventional long-course chemoradiotherapy (CRT) or short-course radiation. Long-course chemoradiotherapy (LCRT) is the conventional method, and it includes 45–54 Gy in 25–28 fractions along with concomitant radio-sensitizing chemotherapy followed by surgery in 6–8 weeks, before or after chemotherapy as part of total neoadjuvant treatment. LCRT is mainly applied in the United States and several European countries [61]. Short-course chemoradiotherapy (SCRT) is composed of total 25 Gy in 5 fractions. SCRT was first used in Northern Europe, with the advantages of reduced cost, improved convenience, and better compliance [62].
- *TNT* TNT includes both CRT and systemic chemotherapy (rather than CRT alone) followed by adjuvant treatment as necessary [63]. It is considered effective in the treatment of micrometastasis. Studies have shown a higher rate of complete response with TNT compared to CRT alone [64]. The advantages include less toxicity, better compliance, and increased local tumor regression, pathologic complete response, and R0 resection rates. Potential pitfalls include overtreatment and unnecessary toxicity in low- or intermediate-risk rectal cancer. It may be performed as induction chemotherapy, when performed before CRT, or consolidation chemotherapy, when performed after CRT [48, 65].
- *Transanal local excision (TAE)* TAE is a local excision surgery characterized by full thickness excision of the tumor down to the mesorectal fat, under direct transanal visualization [66]. There is a potential risk of residual tumor due to the limited field of view. Lymph nodes are not removed and are not pathologically staged. It may be considered for selected patients with early rectal cancer, specifically T1, less than 30% of bowel circumference, less than 3 cm in size, with feasibility of clear margin (> 3 mm) within 8 cm of anal verge [14].
- *Transanal endoscopic microsurgery (TEM/TEMS)* TEM or TEMS is a local excision surgery characterized by full thickness excision of the tumor down to the mesorectal fat, using a rigid rectoscope [66]. This technique provides better tumor exposure and visualization than standard TAE and allows better access to tumors in a higher location. It may be considered for selected patients with early rectal cancer, similar in indication to TAE, with the added benefit that tumor proximal to 8 cm may also be considered if technically feasible. It is associated with less morbidity and a shorter recovery time compared to full oncologic surgery.
- *TAMIS* Like TEMS, TAMIS is used for local excision of early rectal cancer; however, TAMIS utilizes disposable soft device such as silicone proctoscope and does not require the specialized equipment required for TEMS.
- *Watch-and-Wait management* Watch-and-wait management is a potential organ-preserving treatment strategy available to those who achieve complete clinical response after neoadjuvant treatment. It may be referred to as ‘wait and see’ approach. It is appropriate for those who can comply with close surveillance by providers with expertise, as there is potential for tumor regrowth. It is considered a non-standard approach; however, it has been gaining wider acceptance, in part due to an increase in use of TNT, by which up to 50% patients may achieve complete clinical response. The current recommended surveillance includes rectal MRI every 6 months for at least 3 years, and proctoscopy every 3 or 4 months for 2 years, followed by every 6 months up to 5 years. In those with complete clinical response, approximately 25% of patients will experience regrowth, and 10% will experience distant metastasis [67].

## Conclusion

This paper summarizes the SAR Colorectal and Anal Cancer DFP’s revisions and updates to the previously published 2019 rectal cancer imaging and reporting lexicon, incorporating data from newer published clinical and radiologic rectal cancer literature. The primary goal of the lexicon continues to be to improve and enhance efficiency and accuracy in imaging interpretation and communication between radiologists, pathologists, surgeons, oncologists, and patients. Standardized terminology and descriptors are crucial for primary locoregional rectal tumor staging in guiding appropriate multidisciplinary team approach and treatment planning.
